# Impact of vitamin D on cardiac structure and function in chronic kidney disease patients with hypovitaminosis D: a randomized controlled trial and meta-analysis

**DOI:** 10.1093/ehjcvp/pvz080

**Published:** 2019-12-12

**Authors:** Debasish Banerjee, Nihil Chitalia, Irina Chis Ster, Evan Appelbaum, Ravi Thadhani, Juan Carlos Kaski, David Goldsmith

**Affiliations:** 1 Renal and Transplantation Unit, St George’s University Hospital NHS Foundation Trust, G 2.113, Grosvenor Wing, Blackshaw Road, Tooting, London SW17 0QT, UK; 2 Cardiology Clinical Academic Group, Molecular and Clinical Sciences Research Centre, St George’s University of London, London, UK; 3 Renal Medicine, Darent Valley Hospital, Dartford, Kent, UK; 4 Institute of Infection and Immunity, St George's University of London, London, UK; 5 Beth Israel Deaconess Medical Centre, Boston, MA, USA; 6 Department of Biomedical Sciences, Cedars-Sinai Medical Center, Los Angeles, CA, USA; 7 Department of Medicine, Cedars-Sinai Medical Center, Los Angeles, CA, USA

**Keywords:** Chronic kidney disease, Cardiovascular disease, Vitamin D, Cholecalciferol, Left ventricular mass

## Abstract

**Aims:**

Vitamin D deficiency is associated with cardiovascular events in chronic kidney disease (CKD) yet the impact of supplementation is controversial. Previous active vitamin D supplementation studies did not show improvement in cardiac structure or function but the effect of native vitamin D supplementation in CKD patients with low vitamin D levels is unknown. We have addressed this question via both a randomized double-blind prospective study and a meta-analysis of three randomized placebo-controlled studies.

**Methods and results:**

We conducted a randomized double-blind, placebo-controlled trial of vitamin D supplementation in stable, non-diabetic, CKD three to four patients with circulating vitamin D <75nmol/L, who were receiving treatment with ACEi or ARB and had high-normal left ventricular (LV) mass. Patients were randomized to receive six directly observed doses of 100 000 IU cholecalciferol (*n* = 25) or matched placebo (*n* = 23). The primary endpoint was changed in LV mass index (LVMI) over 52 weeks, as assessed by cardiac magnetic resonance imaging. Secondary endpoints included changes in LV ejection fraction (LVEF); LV and right ventricular volumes and left and right atrial area. Vitamin D concentration increased with the administration of cholecalciferol. The change in LVMI with cholecalciferol [median (inter-quartile range), −0.25 g (−7.20 to 5.30)] was no different from placebo [−4.30 g (9.70 to 2.60)]. There was no difference in changes of LVEF; LV and right ventricular volumes and left and right atrial area. The meta-analysis of three 52-week, randomized placebo-controlled studies using active/native vitamin D supplementation showed no differences in LVMI measurements.

**Conclusion:**

Vitamin D supplementation does not have beneficial effects on LV mass in CKD patients.

## Introduction

Adverse cardiovascular (CV) events are the most common cause of morbidity and mortality in patients with chronic kidney disease (CKD). On this basis, many interventions have been deployed to try to improve CV risk in CKD, though no clear successes can be reported.^1^ Vitamin D deficiency is common in CKD and is caused by deficiency of serum 25 hydroxy vitamin D and by decreased activation of this vitamin D species to 1,25-dihydroxy-vitamin D. Observational studies have suggested that CV and renal outcomes in CKD patients are worse in CKD patients with low serum vitamin D concentrations; while some clinical outcomes may possibly improve with vitamin D supplementation.^2–5^ Left ventricular hypertrophy (LVH) a significant risk factor for CV events, such as heart failure and sudden cardiac death, is also very common in CKD.^6^ The LVH in animal experiments is associated low vitamin D activity and can be shown to reverse or improve with supplementation.^7^ In patients with heart failure, vitamin D supplementation given as cholecalciferol 4000 IU/day is associated with improvement ejection function and left ventricular (LV) volume.^8^

There are only two previous randomized trials examining the impact of vitamin D supplementation on LVH in CKD patients. Both of these two previous CKD studies used active vitamin D in the form of paricalcitol (19-nor-1,25-dihydroxy-vitamin D_2_) to try to ameliorate LVH.^9^^,^^10^ The choice of paricalcitol was made in part because of a belief that it would be more effective than a native form of vitamin D, cholecalciferol, which requires enzymatic activation in both the liver and the kidney. The results of the two previous LVH regression studies in CKD were entirely negative, failing to show any benefits in terms of the chosen primary clinical endpoints; moreover, in both studies, significant hypercalcaemia was induced when using paricalcitol, necessitating dose reduction or drug cessation. What is potentially relevant, however, is that native vitamin D as cholecalciferol can be activated by one-alpha-hydroxylase enzyme present in non-renal tissues including the heart and blood vessels, and in the experimental animal model setting this treatment can be shown to reduce intra-cardiac inflammation, renin activity and metalloproteinase inhibition.^11–13^ We have previously demonstrated clinically relevant benefits in arterial endothelial function and stiffness following oral supplementation of cholecalciferol 600 000 units, without any significant treatment-related hypercalcaemia, in one non-randomized and one randomized trial of CKD patients with hypovitaminosis D.^14^^,^^15^ The novel aspects of the proposed study included not only the therapy with cholecalciferol, which was directly observed, but also carefully including patients of mild LVH amenable to change in response to therapy and excluding patients with diabetes avoiding the confounding effects of diabetes on the heart.

The impact of cholecalciferol on cardiac structure and function has not been investigated in the context of CKD in detail.^16^ We studied the effect of cholecalciferol supplementation on the extent and severity of LVH, and on LV function, using cardiac MRI in stable mild to moderate, non-diabetic CKD patients with low vitamin D. We also proceeded to perform a meta-analysis of the three vitamin D treatment trials in CKD to search further for any signal of clinical benefit.

## Methods

The study was a multicentre, parallel arm, double-blind, placebo-controlled trial with patients recruited from St Georges, Guys, Kingston and Kings College hospitals in London, UK. The study was approved by the Surrey Ethics Research Committee (11/H1109/12).

### Study population

Patients were screened if they had stable CKD stages 3 and 4, serum vitamin D concentrations <75 nmol/L, serum calcium concentration <2.5 mmol/L, not on vitamin D therapy, and on chronic ACE inhibitor or angiotensin receptor blocker therapy. Patients with known diabetes, congestive heart failure, valvular heart disease, vasculitis, cancer, active inflammation and on any other form of vitamin D supplementation were excluded. A pre-inclusion screening echocardiogram was performed on all potentially suitable subjects (as above), and patients were then considered fully eligible for the trial if their LV mass index (LVMI) was 80–140 g/m^2^ for females and 100–160 g/m^2^ for males (indicative of the presence of mild LVH).

### Randomization

Patients were block randomized in 1:1 ratio by the research pharmacy team, according to a sequence generated by a study statistician. The research team and patients were unaware of all treatment allocations. The vitamin D and placebo were first stored then dispensed from identical containers throughout the study.

### Study drug administration and follow-up

Each patient was followed up for each vitamin D administration in person. At this visit, the patients’ blood pressures were recorded and their medications reviewed. The study drug or placebo was administered by the research nurses as directly observed 100 000 IU doses (as five capsules of 20 000 IU or five capsules of placebo) at the study visits on weeks 0, 4, 8, 12, 24 and 42 (making a total of 600 000 IU of vitamin D or placebo). Relevant blood tests were done at baseline and at 24 and 52 weeks. The MRI scan was done at baseline and 52 weeks. The participants’ general practitioners were informed that they were participating in a trial, but not what their treatment allocation were. All trial participants and GPs were strongly advised to avoid starting any vitamin D supplementation while in the trial. The patients continued with their regular visits with own nephrologists for their routine clinical care, and again, no vitamin D-based therapy was permitted. As the principal trial endpoint was any impact of vitamin D on LV mass (LVM), cardiac MRI was performed at baseline and then again at 52 weeks.

### Patient safety

All patients were seen by research nurses during vitamin D dose administration and all concerns were addressed. The independent data safety monitoring board (DSMB) reviewed interim blood reports and all reported adverse events.

### Cardiac MRI protocol

All patients were imaged on a 1.5 T Philips Intra MRI scanner with dedicated 32 channel cardiac coil. The protocol included a plane scan, SENSE reference scan, interactive scan to identify four chamber, two chamber long-axis and short-axis geometries of the heart. Breath-holding steady-state free procession 4 chamber, 2 chamber and short-axis stack (1.5, 1.5, 10 mm) cine (30 cardiac phases) were acquired. All images were analysed by single observer blinded to patient treatment allocation on a viewforum work station.

### Sample size determination

The inter-study variability of LVM on cardiac MRI (CMR) measurement in a previous study, using a protocol implemented in the present study, was 2.8–4.8% with a standard deviation of 8.4 g.^17^ Based on these data, we calculated that to demonstrate an LVM difference of 10 g, with α = 0.05 and power 95%, 19 patients were needed in each arm. Previous studies have demonstrated a 10 g difference in LVM with ACEi/ARB therapy using CMR with 10–15 patients.^18^^,^^19^ The two previous studies PRIMO and OPERA of vitamin D therapy in CKD patients considered the 10 g difference on CMR to be acceptable.^9^^,^^10^

Hence to investigate an LVM difference of 10 g with vitamin D therapy the study needed 19 patients in each arm i.e. 38 patients to complete the study (α = 0.05 and power 95%). We expected a dropout rate of 30% and, thus, we had planned to recruit 25 patients to each group.

To test the variability of CMR measured LVM, five patients at baseline were scanned twice using the same CMR protocol. Patients were made to get up from the CMR scanner and then prepared for scanning again. Scans were acquired 10 min apart. The analysis was performed by the same observer twice. This showed the following: LVM measurement on first scan was: 108.52 ± 30.52 g (mean ± SD), LVM measurement on second scan was: 108.43 ± 31.74 g; difference of mean 0.09 g; standard deviation of mean difference 1.53 g. Thus in our hands, CMR thus was a highly reproducible technique for LVM measurement and our original sample size calculation based on an SD of 8.4 g was sufficient to give us a confirmatory result of a 10 g reduction in LVM with vitamin D administration.

### Statistical methods

Descriptive statistics and graphics were used to understand the collected variables, their nature and the appropriateness of the subsequent tests and analyses. Continuous variables are summarized by their mean, standard deviations, inter-quartiles, ranges whilst categorical data by proportions. Parametric or non-parametric tests for independent samples have been applied upon normality assumptions of the variables. For the main clinical markers [left ventricular ejection fraction (LVEF), left ventricular end-diastolic mass (LVED mass), left ventricular end-systolic mass (LVES mass) and left atrial area (LA area)] further detail analyses are presented using ANCOVA and the analysis of change from the baseline.^20^

The current analyses are per-protocol or complete data analyses in modern statistical context. The intention-to-treat analyses or observed data analyses include all participants and are conducted based on missing at random assumption for incomplete observations. Sensitivity analyses are also conducted by setting the missing observations to their minimum/maximum values or carrying the last observation forward. These scenarios are particular single settings and are less than the general analysis above based on missing at random assumption.

### Method of meta-analysis

We conducted a random-effects meta-analysis of all available randomized placebo-controlled studies which investigate the impact of vitamin D (active and nutritional) on LV hypertrophy to test if there is existing evidence for vitamin D therapy to improve LVH in CKD. We conducted an Ovid Medline 1946–2018; Embase 1980–2018 search for randomized controlled studies using the following terms: ‘vitamin D’, ‘left ventricular hypertrophy’, ‘kidney disease’ or ‘chronic kidney disease’. We excluded trials on end-stage kidney disease. We searched the articles by cross-referencing the identified studies. We found two studies both using paricalcitol. Contour enhanced funnel plots for publication bias are also displayed (Supplementary material online)

## Results

### Study subjects

**Figure 1:**
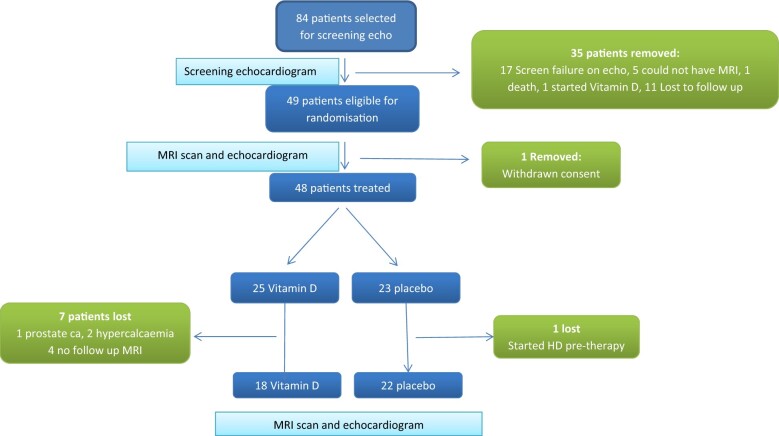
Process of screening, randomization and treatment.

Eighty-four participants underwent a screening echocardiogram. None had significant valvular heart disease, and only one patient had significant regional wall motion abnormality. Sixty-three patients were suitable for recruitment (by LVM measurements). Forty-nine patients were randomized. One patient withdrew from the study (*Figure 1* presents the flow chart of the experiment). Hypertension was commonest cause of CKD, in patient 6 on placebo and patient 9 on vitamin D. Four patients suffered polycystic kidney disease in the placebo group and one in the vitamin D group. Glomerulonephritis was the cause of CKD in five patients on placebo and one patient on vitamin D.

### Cardiac changes

*Table 1* presents summary data at both baseline and follow-up. The baseline comparisons between groups suggest that the randomization produced well-balanced groups. There were no differences in SBP or DBP at baseline or end of study. We do not interpret the *P*-value obtained for the baseline LVEF (*P* = 0.031) as strong evidence against the equality of the means across treatment groups. Preliminary tests for the follow-up values have also been presented and no strong evidence against the null hypothesis of no differences between the intervention groups was found.

**Table 1:** Summary of the population data: demographics, cardiovascular risks and relevant clinical characteristics

	Placebo (*N* = 23)			Vitamin D (*N* = 25)			
	Mean (SD)	Median (Q1, Q3)	Number	Mean (SD)	Median (Q1, Q3)	Number (N)	*P*-value
Demographics							
Age	52.4 (10.9)	51 (45, 61)	23	52.4 (12.5)	52 (42, 62)	25	0.94
Gender—female			9 (39%)			7 (28%)	0.41
Ethnicity—Caucasian			14 (60.1%)			12 (48%)	0.46
Black			6 (26.1%)			7 (28%)	
Others			1 (4.4%)			4 (16%)	
Missing			2 (8.7%)			2 (8%)	
Cardiovascular risk							
Hypertention (yes)			23 (100%)			23 (92%)	0.49
Smoking (no)			21 (91%)			21 (88%)	0.61
BMI (kg/m^2^)	29 (4.7)	28 (25, 33)	23	29 (4.11)	29 (27, 31)	25	0.76
Systolic blood pressure	133.7 (14.4)	133 (120, 139)	22	144 (22.8)	140 (127, 157)	23	0.09
Diastolic blood pressure	85.2 (11.9)	82 (78, 88)	22	90.1 (13.8)	89 (79, 100)	23	0.21
Laboratory values							
Calcium (mmol/L)	2.37 (0.11)	2.35 (2.29, 2.44)	22	2.4 (0.11)	2.38 (2.36, 2.49)	20	0.37
Phosphate (mmol/L)	1.07 (0.29)	1.1 (1, 1.2)	22	1.15 (0.17)	1.14 (1, 1.3)	20	0.42
Parathyroid hormone (pmol/L)	10.5 (6.9)	9 (6.8, 11.7)	20	12.1 (9.4)	8.9 (5.8, 14.9)	16	0.90
Vitamin D screening (nmol/L)	44.5 (20.45)	49.5 (29, 64)	18	42.75 (17.83)	39.50 (28.50, 59.50)	20	0.95
Vitamin D baseline (nmol/L)	45.95 (26.24)	49 (19.5, 64.5)	20	36.67 (17.41)	35 (22, 47)	15	0.22
Haemoglobin (g/dL)	12.84 (1.03)	12.75 (12.1, 13.8)	22	12.89 (1.39)	12.8 (12.2, 13.6)	21	0.90
Creatinine (µmol/L)	190.13 (72.29)	169 (134, 237)	23	186.57 (51.34)	173 (149, 218)	21	0.72
eGFR (ml/min/1.73m^2^)	34.19 (11.25)	36 (25, 41)	21	35.32 (10.92)	36 (26, 41)	19	0.75

The *P*-values correspond to appropriate two-independent sample tests (χ^2^, *t*-test or Kruskal–Wallis) which evaluated the null hypotheses of no difference between the two treatment groups.

**Figure 2:**
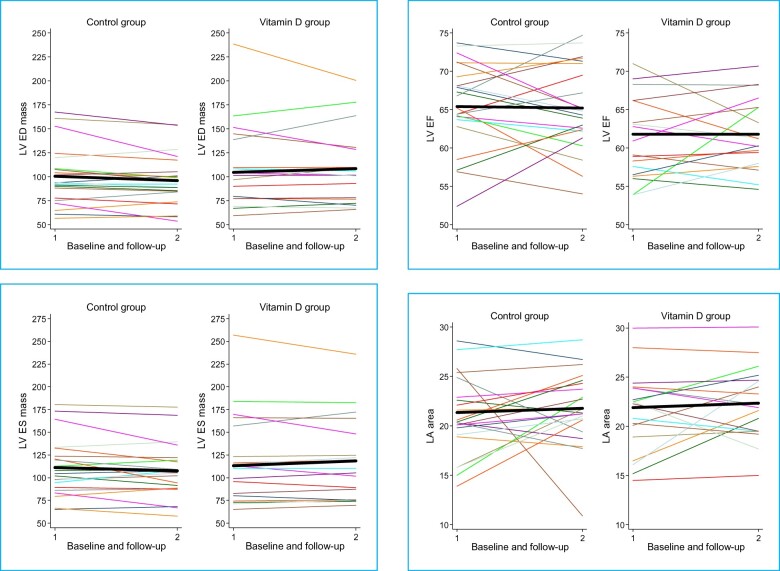
Raw data representing individual variability for the main cardiac variables. The black thick line joins the raw mean at the baseline (1) with that at the follow-up (2).

However, to understand the full extent of the differences between groups at the follow-up and how they changed over the intervention period with regards to the four clinical markers, ANOVA and the analyses of the changes are carried out. Results are presented in Supplementary material online, *Tables S5 and S6*; *Tables 2 and **3* and graphical representation of these results is presented in *Figure 2*.

**Table 2:** Change from the baseline raw data summary for clinical outcomes of interest

	Placebo (*N* = 23)			Vitamin D (*N* = 25)			
	Mean (SD)	Median (Q1, Q3)	Number	Mean ( SD)	Median (Q1, Q3)	Number	Test
Change LVED mass (g)	−4.54 (9.75)	−4.30 (−9.70, 2.60)	22	−1.76 (14.03)	−0.25 (−7.20, 5.30)	18	0.47
Change LVESV (ml)	1.60 (10.40)	4.10 (−2.80, 6.70)	22	−1.76 (8.29)	−2.95 (−5.50, 4.00)	18	0.26
Change LVEDV (ml)	1.77 (21.13)	1.00 (−12.20, 12.70)	22	−1.56 (14.11)	−5.85 (−10.30, 4.80)	18	0.57
Change LVEF (%)	−0.37 (4.88)	−1.50 (−3.60, 3.80)	22	0.61 (4.25)	0.90 (−2.00, 2.10)	18	0.51
Change LA area (cm^2^)	0.79 (4.79)	1.05 (−1.30, 4.20)	22	0.88 (3.36)	0.20 (−1.30, 3.60)	18	0.51
Change RVEDV (ml)	4.18 (23.38)	2.45 (−5.40, 17.00)	22	3.37 (36.05)	0.15 (−13.80, 14.20)	18	0.65
Change RVESV (ml)	−1.30 (16.14)	0.65 (−12.80, 6.60)	22	−7.90 (36.77)	1.95 (−12.20, 6.20)	18	0.96
Change RVSV (ML)	6.64 (16.18)	5.50 (3.70, 18.20)	22	3.46 (18.04)	1.35 (−4.80, 4.60)	18	0.09
Change RVEF (%)	2.58 (5.96)	3.70 (−3.40, 7.20)	22	0.06 (7.34)	2.25 (−6.10, 4.10)	18	0.25
Change RA area (cm^2^)	−0.05 (3.61)	−0.50 (−1.40, 2.50)	22	1.30 (2.92)	1.45 (−1.50, 3.00)	18	0.21

The *P*-values correspond to appropriate two-independent sample tests (*t*-test or Kruskal–Wallis after checking the normality assumption) which evaluated the generic null hypotheses of no difference between the samples’ distributions corresponding to the two treatment groups. EF, ejection fraction; ESV, end systolic volume; LA, left atrial; LVED, left ventricular end-diastolic; RA, right atrial.

**Table 3:** Results from ANCOVA and the analysis of the change from the baseline models

Outcome	Estimate	Standard error	*z*	*P*-value	95% CI low	95% CI high	No. obs.	Shapiro test
The analysis of tde follow-up values								
Follow-up LVEF (%)								
Baseline LVEF	0.6	0.12	4.94	<0.001	0.36	0.85	40	0.5
Vit. D vs. placebo	−0.78	1.41	−0.55	0.586	−3.64	2.09		
Constant	65.11	0.88	74.14	<0.001	63.33	66.89		
Follow-up LVED mass (g)								
Baseline LVED mass	0.84	0.05	18.40	<0.001	0.75	0.93	40	0.02
Vit. D vs. placebo	4.33	3.35	1.29	0.204	−2.46	11.12		
Constant	95.55	2.23	42.90	<0.001	91.03	100.06		
Follow-up LVES mass (g)								
Baseline LVES mass	0.9	0.04	23.70	<0.001	0.82	0.98	40	0.55
Vit. D vs. placebo	3.3	3.05	1.08	0.285	−2.87	9.48		
Constant	107.14	2.03	52.69	<0.001	103.02	111.26		
Follow-up LA area (cm2)								
Baseline LA area	0.49	0.11	4.44	<0.001	0.27	0.71	39	0.24
Vit. D vs. placebo	−0.29	0.87	−0.33	0.742	−2.05	1.47		
Constant	22.41	0.59	38.09	<0.001	21.22	23.6		
The analysis of the change from the baseline								
Change in LVEF (%)								
Baseline LVEF	−0.4	0.12	−3.26	0.002	−0.64	−0.15	40	0.50
Vit. D vs. placebo	−0.78	1.41	−0.55	0.586	−3.64	2.09		
Constant	−0.29	0.88	−0.33	0.740	−2.07	1.49		
Change in LVED (ml)								
Baseline LVED mass	−0.16	0.05	−3.46	0.001	−0.25	−0.07	40	0.023
Vit. D vs. placebo	4.33	3.35	1.29	0.204	−2.46	11.12		
Constant	−4.45	2.23	−2.00	0.053	−8.97	0.06		
Change in LVES mass (g)								
Baseline LVES mass	−0.23	0.04	−5.85	<0.001	−0.31	−0.15	40	0.72
Vit. D vs. placebo	6.13	3.2	1.92	0.063	−0.34	12.61		
Constant	−15.08	2.13	−7.07	<0.001	−19.4	−10.76		
Change in LA area (cm2)								
Baseline LA area	−0.51	0.11	−4.62	<0.001	−0.73	−0.29	39	0.24
Vit. D vs. placebo	−0.29	0.87	−0.33	0.742	−2.05	1.47		
Constant	1.41	0.59	2.40	0.022	0.22	2.6		

Shapiro tests are used to assess models’ appropriateness, i.e. normality of the residuals. EF, ejection fraction; ESV, end systolic volume; LA, left atrial; LVED, left ventricular end-diastolic; RA, right atrial.

Based on observed data analysis, in essence intention-to-treat analysis (using the available information which excludes the loss at the follow-up) no difference in LVM or LVEF, between the groups was observed, when adjusted for the corresponding baseline measurements with regards to either follow-up or the change seen in the trial. The specific interpretation of the results regarding LVEF is given below.

The evidence suggests that the follow-up LVEF is linked with the baseline LVEF, namely for every one unit increase in baseline LVEF, there is a 0.6 unit increase in follow-up LVEF (*P* < 0.001) and there is no evidence to support a difference between the two intervention groups (*P* = 0.586, *Table 3*). The constant (65.11) represented the estimated mean of the follow-up LVEF in the placebo group.

The change in LVEF decreases with increasing baseline LVEF (*P* = 0.002) and there is no evidence to suggest that this is different across intervention groups (*P* = 0.59). The constant (−0.29) represents the average change in the LVEF in the Placebo group.

Both follow-up and changes in the clinical outcomes of interest are explained by their respective baseline measurements, as expected (*Table 3*). However, the adjusted effect of the intervention did not reveal significant differences between the two groups. Intention-to-treat analyses results do not differ from the above; neither in terms of the magnitude of the estimates nor in terms of their precision. For more information, please see Supplementary material online, *File text* and *Stables A–D*. Sensitivity analyses to the missing data did not suggest a different assembly picture of the results.

There was no difference in MRI measured LV and RV volumes and RV function, LA and right atrial areas at the end of the study between active and placebo groups (see Supplementary material online, *Table S6*).

### Biochemical parameters

**Figure 3:**
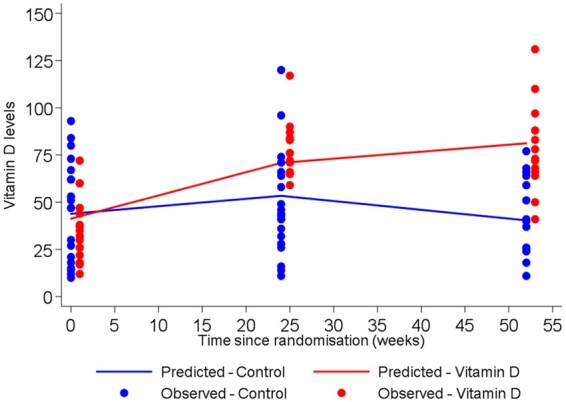
25 hydroxy vitamin D concentration in patients on vitamin D and placebo at baseline, 24 and 52 weeks.

Analysis of data suggests a quadratic trend with time (*P* < 0.001) for the Vitamin D levels in the two groups. However, this quadratic trend differs amongst the two clinical groups with increasing trend in vitamin D group and decreasing levels in placebo group after 24 weeks (*P* < 0.001; *Figure 3*). The predicted (and observed) levels of vitamin D as shown in the figure correspond to a mixed model which correctly accounts for the longitudinal structure of the data.

Systolic blood pressure did not differ in the vitamin D vs. the placebo group, at baseline [133.7 (14.4) vs. 144 (22.8) mmHg; *P* = 0.09] and end of study [131.4 (16.0) 131.6 (14.4) mmHg; *P* = 0.82]. eGFR at baseline was not different between the vitamin D and placebo group [34.19 (11.25) vs. 35.32 (10.92 mL/min/1.73m^2^); *P* = 0.75]. There was no difference between vitamin D and placebo groups in creatinine concentrations at baseline [197 (68) vs. 189 (61) µmol/L; *P* = 0.70], 24 weeks [194 (63) vs. 187 (75) µmol/L; *P* = 0.75) and 52 weeks [219 (103) vs. 190 (86) µmol/L; *P* = 0.42]. Moreover, there was no difference in creatinine changes over time within the two groups. There were no differences between vitamin D or placebo groups at 52 weeks for calcium, phosphate or parathyroid hormone concentrations. None of the patients experienced cardiac events during the study period. 

### Meta-analysis

**Figure 4:**
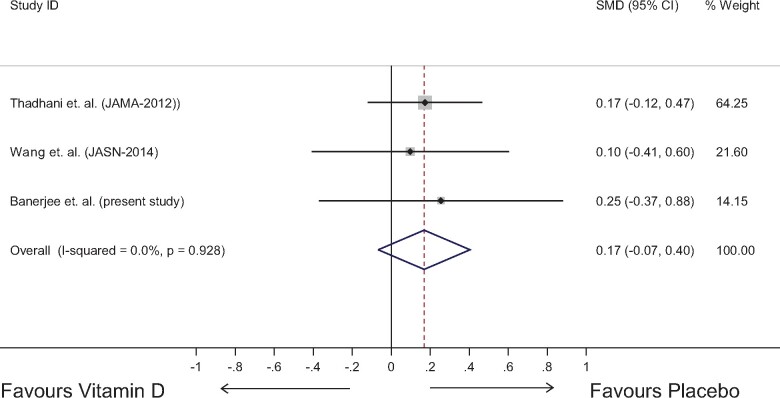
Meta-analysis of studies on the impact of vitamin D on left ventricular mass in CKD patients. SMD, standard mean difference.

Three randomized clinical trials, including the current study (*Table 4*), which investigated the effect of the intervention on changes in LVMI have been collated and a random-effects meta-analysis has been conducted for to derive a pooled group effect. The studies did not exhibit much heterogeneity (*I*^2^ = 0.0%) and hence the pooled estimate, based on a larger numbers (*Figure 4*) can be interpreted. The analysis suggests that the changes favour placebo, with a standardized mean difference of 0.17 (-0.07, 0.40) but the difference is not statistically significant. The result suggests that the data are consistent with no difference in the LVMI between the two treatment groups.

**Table 4:** Meta-analysis of randomized trials on impact of vitamin D on left ventricular mass in CKD patients

Study	Vitamin D			Placebo		
	Number	Mean (LVMI g/m^2.7^)	95% CI	Number	Mean (LVMI g/m^2.7^)	95% CI
Thadhani *et al.*	88	0.34	−0.15 to 0.83	91	−0.07	−0.56 to 0.42
Wang *et al.*	30	−1.75	−3.69 to 0.19	30	−2.28	−4.22 to 0.23
Banerjee *et al.* (present study)	22	−0.33	−2.84 to 2.18	18	−1.00	−2.62 to 0.62

Studies used in meta-analysis—showing the number of patients, mean change in LVMI.

This is a relatively small meta-analysis—perhaps suggesting that more research is needed to understand the true intervention effect. We conducted a fixed-effect meta-analysis and there was no difference between fixed-effect and random-effect meta-analyses. We have decided to conduct a further patient-level data meta-analysis with additional clinical outcomes including renal function. The funnel plot (a scatter plot of the effect estimates from individual studies against some measure of each study's size or precision) is shown in the Supplementary material online, *Figure S1*.

### Adverse events and the course of the study

During the trial, one patient developed prostate cancer and was withdrawn from study (this was not considered to be a treatment-emergent event). One patient suffered rapid progression of kidney disease and ended up on haemodialysis and hence withdrawn from study (this was not accompanied or caused by hypercalcaemia). Two patients randomized to vitamin D developed mild biochemical hypercalcaemia and the data safety monitoring board requested breaking of randomization code and for them to be withdrawn from study. One patient was subsequently diagnosed with granulomatous disease and the other developed primary hyperparathyroidism. One patient failed to attend the study appointments and requested to be withdrawn from the study. There was no issue reported by patients during directory observed therapy with vitamin D or placebo.

Patients who were suitable by echocardiographic criterion for randomization were not all randomized to treatment due various reasons such as inability to underdo MRI due to claustrophobia and metallic clips (5), rapid progression of kidney disease (1), death due to pulmonary embolism (1) and inability to schedule follow-up visits (3).

The patient flow is described in *Figure 1*, including screening, randomization and treatment-emergent outcomes.

## Discussion

The present study of stable CKD patients with hypovitaminosis D showed that treatment with 600 000 units of cholecalciferol over a 52 weeks period did not change LV structure or function, compared with placebo, despite biochemical evidence of a significant and sustained rise in serum 25(OH)D concentrations in the active-treated group. There was no difference in MRI measured LVM, LV function, right ventricular structure, right ventricular function, right atrial and LA area, between subjects randomized to vitamin D repletion, or placebo.

The results of this study are very similar (i.e. negative) to the previously conducted randomized trials of active vitamin D therapy in CKD patients namely ‘PRIMO’ and ‘OPERA’.^9^^,^^10^

It is instructive to compare these three trials which have attempted to use vitamin D therapy to alter cardiac structure and function in CKD patients (these three studies are the only ones to have been conducted in CKD to date). This comparison can be seen in part in *Table 5*.

**Table 5:** Comparison of the trials vitamin D therapy to improve left ventricular mass in CKD patients

	PRIMO (*n* = 227)	OPERA (*n* = 60)	5C study (*n* = 48)
Baseline characteristics			
Population studied	Pre-dialysis CKD stage 3, 4; multinational	Pre-dialysis CKD stages 3, 4, 5; Chinese	Pre-dialysis CKD stages 3, 4; multi-ethnic
LV characteristics	Mild or no LVH	Moderate LVH (LVMI 70% or higher, diastolic stiffness)	Mild LVH (LVMI F 80–140 M 100–160 g/m^2^)
ACEi/ARB	95%	85%	100%
Diabetes included	Yes	Yes	No
Vitamin D concentration	Not measured	Not measured	43 nmol/L
Therapy			
Intervention	Paricalcitol	Paricalcitol	Cholecalciferol
Duration of therapy	48 weeks	52 weeks	40 weeks
Dose of Vitamin D	2 µg/d	1 µg/d	100 000 IU six doses
Effects			
Parathyroid hormone changes	Significant decrease	Significant decrease	Mild decrease
Hypercalcaemia (%)	23%	44%	2/49 (4%)
LVMI	No change	No change	No change
Hospitalization	Lower hospitalization^a^	Lower hospitalization^a^	—
Other effects	Low BNP; low LA volume		

a Not powered but pre-specified endpoint.

LA, left atrial; LVH, left ventricular hypertrophy.

### PRIMO

Participants in PRIMO were randomly assigned to receive oral paricalcitol or placebo. The primary outcome measure was the change in LVMI over 48 weeks assessed by CMR. Secondary end points included echocardiographic changes in LV diastolic function. The results showed that treatment with paricalcitol significantly reduced PTH concentration within 4 weeks and maintained this suppression to within the normal range throughout the study duration. While at 48 weeks, the change in LVMI did not differ between treatment groups [paricalcitol group, 0.34 g/m^2.7^ (95% CI, −0.14, 0.83 g/m^2.7^) vs. placebo group, −0.07 g/m^2.7^ (95% CI, −0.55, 0.42 g/m^2.7^)]. Doppler measures of diastolic function including peak early diastolic lateral mitral annular tissue velocity [paricalcitol group, −0.01 cm/s (95% CI, −0.63, 0.60 cm/s) vs. placebo group, −0.30 cm/s (95% CI, −0.93, 0.34 cm/s)] also did not differ. More adverse events were judged to be probably or possibly drug related primarily due to hypercalcaemia (paricalcitol, 22.6% vs. placebo, 0.9%; *P* < 0.001).

### OPERA

The primary endpoint of this study was changed in LVM indexed by body surface area or height2^.7^ after 52 weeks of oral paricalcitol or placebo, which again did not differ between groups at the end of the study period. Several secondary endpoints, including change in other pre-specified CMR parameters, such as LV volume index, did not differ either; their included LVEF and other echocardiographic parameters, ratio of early to late transmitral inflow velocity (E/A), tissue Doppler-derived measure of Eʹ, late diastolic mitral annulus velocity, systolic mitral annulus velocity and ratio of E/Eʹ. The most significant side-effect attributable to paricalcitol treatment was hypercalcaemia with significant suppression of PTH.

### Meta-analysis

We then conducted a meta-analysis of all three studies examining the impact of vitamin D treatment on LVM in CKD. As stated, our main findings were similar to and confirmatory of the two previous trials of vitamin D in mild-to-moderate LVH in CKD.

Despite the reported outcome similarities (no measurable impact on LV structure or function), there were some potentially important differences between these three studies. One of the key differences was around the selection of potential patients for inclusion.

In the PRIMO study, septal wall LV thickness alone was used as one of the inclusion criteria, whereas in our ‘5C’ study, and in the OPERA study, patient inclusion was based on standard echocardiographic criteria of LV hypertrophy. The LVMI of OPERA subjects was at least 70% higher than the LVMI in the PRIMO study and the 5C study. A major reason suggested for the negative findings in the PRIMO study was the low LVM and absence of marked LV hypertrophy, and this might well have been the case for the 5C study as well. All three studies clearly showed that vitamin D treatment had no demonstrable effect on reducing LVM over approximately 52 weeks. Furthermore, a large proportion of all three sets of study subjects exhibited diastolic dysfunction at baseline, which again was unaffected by the administration of vitamin D. Another important conceptual difference between our ‘5C’ study and the two previous studies was our screening of potential participants by their serum vitamin D [25(OH)D] concentrations, selecting only those subjects with insufficient circulating vitamin D. Thus, we would have avoided treating patients with no measurable vitamin D deficiency. It could be argued that the elevation of serum PTH was a manifestation of functional vitamin D deficiency in both PRIMO and OPERA but we feel that this is a moot point. In our ‘5C’ study, we did not choose to randomize potential trial subjects with absolute vitamin D deficiency, feeling that it was ethically preferable to replete such patients with vitamin D. It is certainly true that all three studied patient cohorts had significantly elevated serum PTH concentrations—a functional definition of secondary hyperparathyroidism, and likely largely to be secondary to lack of vitamin D activity, and that these PTH concentrations fell very significantly in the PRIMO and OPERA patient cohorts once subjects were exposed to activated vitamin D in the form of paricalcitol (but at the evident cost of significant biochemical hypercalcaemia).

In our 5C study, the pre-repletion serum 25(OH)D concentration was 42 nmol/L and this rose significantly over the course of the year’s trial duration, but only in those subjects administered vitamin D—from baseline values of 42 to 79 nmol/L (*P* < 0.001). In the ‘5C’ study, the impact on serum PTH concentrations was not significant, which is in marked contrast to what was seen in PRIMO and OPERA. Equally, we only saw two cases of hypercalcaemia, probably none related to study drug (native vitamin D). This should be contrasted with two recent studies of native vitamin D repletion, one conducted in subjects with heart failure where LV function did improve, and the baseline serum 25(OH)D concentrations rose from 30 to 90 nmol/L, and another in CKD patients where vasomotor endothelial function improved significantly only in the vitamin D treated cohort, whose baseline serum 25(OH)D rose from 30 to 90 nmol/L.^8^^,^^15^ Interestingly, in these two positive studies, both of which were of shorter duration than ‘5C’, PRIMO or OPERA, there was little to no hypercalcaemia when using native vitamin D (cholecalciferol) as the active intervention.

Another potential explanation for the negative results in all three trials would be that the treatment duration was too short to modify LV hypertrophy and dysfunction; this can only be proved by attempting a longer duration of study, though this would present significant challenges for activated vitamin D compounds which are now discouraged by the latest version of the KDIGO CKD-MBD guidelines on account of the likely cumulative incidence of hypercalcaemia.^21^

Another possibility for the negative results may be that because activated vitamin D acts by repressing the RAS and all of our (‘5C’) patients already received treatment with RAS blockers, the effect of activated D treatment on the myocardium may possibly be attenuated because of concomitant treatment with RAS blockers. This is, of course, true also of PRIMO and especially OPERA where >80% of subjects were chronically exposed to RAS blockade (as remains guideline-mandated therapy for hypertensive proteinuric CKD patients). While studying patients who were not treated with RAS blockers would be interesting, it might be unethical.

In conclusion, there have now been three separate independent studies of the use of vitamin repletion or treatment to reduce LVM and to improve LV function, and all three were negative trials. Although the total number of subjects in all the three trials was only 335, there is no suggestion that this approach to target LVH which is so common in CKD is efficacious. Other approaches, such as the use of RAS blockade, and, in dialysis patients, the use of daily augmented-dose dialysis regimens, appear to be more successful in this regard, though to date without compelling evidence of better patient survival.^22^ For the time being, therefore, the proper use of vitamin D therapy in CKD appears to be to target serum PTH elevation, i.e. using a combined biochemical/skeletal target.

## Supplementary material

Supplementary material is available at *European Heart Journal – Cardiovascular Pharmacotherapy* online.

## Funding

This work was supported by British Heart Foundation to DB-[PG10/71/28462] and partially by Wellcome-ISSF to DB-[204809/Z/16/Z].

**Conflict of interest:** D.G.—lecture and advisory board fees from Amgen, Sanofi, Vifor Fresenius; Dr I.C.S. conducted the statistical strategy of the paper, implemented the analyses, provided their interpretation and contributed to the writing of first draft of the paper; R.T.—consultant for Fresenius Medical Care North America; D.B.—Speaker fees from AstraZeneca, Pfizer, ViforPharma.

## Supplementary Material

pvz080_Supplementary_Data
