# Supplementary material for: Impact of vitamin D on cardiac structure and function in chronic kidney disease patients with hypovitaminosis D: a randomized controlled trial and meta-analysis
Source: Eur Heart J Cardiovasc Pharmacother. 2019 Dec 12;7(4):302–11. doi: 10.1093/ehjcvp/pvz080 (PMC8302255; doi:10.1093/ehjcvp/pvz080)
Supplement: pvz080_Supplementary_Data [file pvz080_supplementary_data.docx]

**Supplementary material** (EHJ-CVP-D-19-00120)

***Descriptive statistics***

Descriptive statistics and graphics were used to understand the collected variables, their nature and the appropriateness of the subsequent tests and analyses. Continuous variables are summarized by their means, medians, standard deviations, inter-quartiles as well as the number of observations the summaries rely on. Summary statistics are presented by intervention group and for both baseline and follow up. Parametric tests such as t-tests were applied when data did not exhibit strong departures from normality and Kruskal-Wallis non parametric tests when the contrary has been observed. Variables departure from normality were assessed using Shapiro-Wilk test with p-values smaller than 0.05 indicating strong evidence against normality. Categorical/binary are summarized as proportions and chi-squared type tests were applied to test the corresponding null hypothesis. P-values less than 0.05 were considered statistically significant and the uncertainty was quantified through standard errors and 95% confidence intervals.

***Analysis***

For the main clinical markers (LV EF, LV ED, LV ES mass and LA area) further detail analyses are presented using ANCOVA and the analysis of change from the baseline. A thorough review of the methods can be found in (1). These models are in essence regression techniques with scores at the follow up or differences between the follow up and the baseline as statistical outcomes and randomisation groups and baseline scores as covariates. Interaction models have also been considered – that is exploring whether the association between the change and the corresponding baseline measurement differ between the groups. The results are not shown as they it appeared to not be the case. Models appropriateness have been assessed by checking residuals’ departure from normality. When this was strongly violated transformations such log have been considered and models refitted and the fit improved. These results are not shown as the qualitative message of the analyses have not changed - the original versions have been presented for simplicity.

Outliers and influential points have been taken out and the models refitted – results are not shown as the results were consistent with those which included all the available information.

The data were analysed as per-protocol (complete data analyses) and on intention-to-treat (ITT) basis. However, patterns in the missing data have been evaluated and sensitivity analyses to the ITT results have been carried out. Assumptions on the missing data were that of missing at random, after investigating patterns in the missing data (Table A below).

***Meta-analysis***

Three similar studies have been combined to derive a pooled estimate for the differences in changes in left ventricular mass across treatment groups. The studies were consistent with not much heterogeneity between them (p=0.93). However, the pooled overall estimate for the mean difference (0.17(95%CI(-0.07, 0.4))) was not deemed as significantly different form 0 (p=0.16) and hence, based on these data, there is not enough evidence to suggest a statistically significant effect of Vitamin D. The analyses were assuming random effects although, on this particular data, there was no difference in the results between that and the results based on fixed effects – a much more restricted framework in terms of assumptions.

Table S1: Potential patterns in the missing data

| **THE ANALYSIS OF MISSING OUTCOMES** | | | | | | | | | |
| --- | --- | --- | --- | --- | --- | --- | --- | --- | --- |
| **Outcome** |  | **OR** | **St Error** | **z** | **p-value** | **95%CI low** | **95%CI low** | **No obs** |  |
| **Follow up LV EF (%)** | **Baseline LV EF** | 1.04 | 0.08 | 0.47 | 0.638 | 0.89 | 1.20 | 47 |  |
|  | **Vit D vs. Placebo** | 5.85 | 5.87 | 1.76 | 0.078 | 0.82 | 41.82 |  |  |
| **Follow up LV ED mass (g)** | **Baseline LV ED mass** | 0.99 | 0.01 | -0.84 | 0.401 | 0.96 | 1.02 | 47 |  |
|  | **Vita D vs. Placebo** | 5.61 | 5.40 | 1.79 | 0.073 | 0.85 | 37.05 |  |  |
| **Follow up LV ES mass (g)** | **Baseline LV ES mass** | 0.99 | 0.01 | -0.66 | 0.507 | 0.97 | 1.01 | 47 |  |
|  | **Vit D vs. Placebo** | 5.50 | 5.27 | 1.78 | 0.075 | 0.84 | 35.95 |  |  |
| **Follow up LA area (cm2)** | **Baseline LA area** | 1.16 | 0.11 | 1.52 | 0.127 | 0.96 | 1.39 | 47 |  |
|  | **Vit D vs. Placebo** | 2.64 | 2.17 | 1.17 | 0.24 | 0.52 | 13.28 |  |  |

Legend: The analyses of missing outcomes on randomisation groups and their respective baseline values. A (penalized maximum likelihood) logistic regression has been applied for a newly created outcome indicating 1 if the follow-up individual values (or the change indeed) is missing and 0 otherwise on randomisation group and the respective baseline value. One missing value at the baseline has been left as such.

Table S2: Sensitivity analysis of the missing baseline value as minimum

| **THE ANALYSIS OF MISSING OUTCOMES – the missing baseline set to its minimum value** | | | | | | | | | |
| --- | --- | --- | --- | --- | --- | --- | --- | --- | --- |
| **Outcome** |  | **OR** | **St Error** | **z** | **p-value** | **95%CI low** | **95%CI low** | **No obs** |  |
| **Follow up LV EF (%)** | **Baseline LV EF** | 1.00 | 0.07 | -0.04 | 0.972 | 0.87 | 1.14 | 48 |  |
|  | **Vit D vs. Placebo** | 5.82 | 5.72 | 1.8 | 0.073 | 0.85 | 39.86 |  |  |
| **Follow up LV ED mass (g)** | **Baseline LV ED mass** | 0.98 | 0.02 | -1.13 | 0.259 | 0.95 | 1.01 | 48 |  |
|  | **Vita D vs. Placebo** | 6.43 | 6.15 | 1.95 | 0.052 | 0.99 | 41.90 |  |  |
| **Follow up LV ES mass (g)** | **Baseline LV ES mass** | 0.99 | 0.01 | -0.93 | 0.35 | 0.97 | 1.01 | 48 |  |
|  | **Vit D vs. Placebo** | 6.27 | 5.96 | 1.93 | 0.053 | 0.97 | 40.41 |  |  |
| **Follow up LA area (cm2)** | **Baseline LA area** | 1.08 | 0.09 | 0.99 | 0.322 | 0.92 | 1.27 | 48 |  |
|  | **Vit D vs. Placebo** | 3.30 | 2.63 | 1.5 | 0.134 | 0.69 | 15.77 |  |  |

Legend: Sensitivity analysis 1: as above but the missing value at the baseline was set up to its respective minimum.

Table S3: Sensitivity analysis with missing baseline value as maximum

| **THE ANALYSIS OF MISSING OUTCOMES – the missing baseline set to its maximum value** | | | | | | | | | |
| --- | --- | --- | --- | --- | --- | --- | --- | --- | --- |
| **Outcome** |  | **OR** | **St Error** | **z** | **p-value** | **95%CI low** | **95%CI low** | **No obs** |  |
| **Follow up LV EF (%)** | **Baseline LV EF** | 1.07 | 0.08 | 1 | 0.316 | 0.93 | 1.23 | 48 |  |
|  | **Vit D vs. Placebo** | 7.61 | 7.53 | 2.05 | 0.04 | 1.10 | 52.84 |  |  |
| **Follow up LV ED mass (g)** | **Baseline LV ED mass** | 1.00 | 0.01 | 0.08 | 0.936 | 0.98 | 1.02 | 48 |  |
|  | **Vita D vs. Placebo** | 6.06 | 5.75 | 1.9 | 0.058 | 0.94 | 38.95 |  |  |
| **Follow up LV ES mass (g)** | **Baseline LV ES mass** | 1.00 | 0.01 | 0.21 | 0.831 | 0.99 | 1.02 | 48 |  |
|  | **Vit D vs. Placebo** | 6.01 | 5.70 | 1.89 | 0.059 | 0.94 | 38.57 |  |  |
| **Follow up LA area (cm2)** | **Baseline LA area** | 1.18 | 0.11 | 1.83 | 0.067 | 0.99 | 1.42 | 48 |  |
|  | **Vit D vs. Placebo** | 2.85 | 2.34 | 1.27 | 0.203 | 0.57 | 14.27 |  |  |

Legend: Sensitivity analysis 2: as above but the missing value at the baseline was set up to its respective maximum.

Table S4: Multiple imputation analysis

| **THE ANALYSIS OF THE FOLLOW UP VALUES** | | | | | | | | | |
| --- | --- | --- | --- | --- | --- | --- | --- | --- | --- |
| **Outcome** |  | **Estimate** | **St Error** | **z** | **p-value** | **95%CI low** | **95%CI low** |  |  |
| **Follow up LV EF (%)** | **Baseline LV EF** | 0.59 | 0.12 | 4.85 | <0.001 | 0.35 | 0.84 |  |  |
|  | **Vit D vs. Placebo** | -0.86 | 1.41 | -0.61 | 0.55 | -3.72 | 2.01 |  |  |
|  | **Constant** | 65.10 | 0.88 | 74.14 | <0.001 | 63.33 | 66.87 |  |  |
| **Follow up LV ED mass (g)** | **Baseline LV ED mass** | 0.84 | 0.05 | 17.95 | <0.001 | 0.75 | 0.93 |  |  |
|  | **Vita D vs. Placebo** | 4.71 | 3.35 | 1.41 | 0.17 | -2.09 | 11.51 |  |  |
|  | **Constant** | 95.43 | 2.26 | 42.26 | <0.001 | 90.87 | 99.99 |  |  |
| **Follow up LV ES mass (g)** | **Baseline LV ES mass** | 0.90 | 0.04 | 23.64 | <0.001 | 0.82 | 0.98 |  |  |
|  | **Vit D vs. Placebo** | 3.22 | 3.05 | 1.05 | 0.30 | -2.98 | 9.41 |  |  |
|  | **Constant** | 107.16 | 2.05 | 52.28 | <0.001 | 103.02 | 111.30 |  |  |
| **Follow up LA area (cm2)** | **Baseline LA area** | 0.48 | 0.11 | 4.36 | <0.001 | 0.26 | 0.71 |  |  |
|  | **Vit D vs. Placebo** | -0.33 | 0.89 | -0.37 | 0.72 | -2.14 | 1.48 |  |  |
|  | **Constant** | 22.38 | 0.60 | 37.02 | <0.001 | 21.16 | 23.61 |  |  |
|  | | | | | | | | | |
| **Change in LV EF (%)** | **Baseline LV EF** | -0.40 | 0.12 | -3.24 | <0.001 | -0.66 | -0.15 |  |  |
|  | **Vit D vs. Placebo** | -0.80 | 1.45 | -0.55 | 0.59 | -3.74 | 2.15 |  |  |
|  | **Constant** | -0.27 | 0.88 | -0.30 | 0.76 | -2.05 | 1.52 |  |  |
| **Change in LV ED (ml)** | **Baseline LV ED mass** | -0.16 | 0.05 | -3.43 | <0.001 | -0.25 | -0.06 |  |  |
|  | **Vita D vs. Placebo** | 4.32 | 3.36 | 1.29 | 0.21 | -2.50 | 11.14 |  |  |
|  | **Constant** | -4.53 | 2.25 | -2.01 | 0.05 | -9.08 | 0.02 |  |  |
| **Change in LV ES mass (g)** | **Baseline LV ES mass** | -0.23 | 0.04 | -5.59 | <0.001 | -0.32 | -0.15 |  |  |
|  | **Vit D vs. Placebo** | 6.34 | 3.29 | 1.92 | 0.06 | -0.35 | 13.02 |  |  |
|  | **Constant** | -15.09 | 2.17 | -6.95 | <0.001 | -19.48 | -10.71 |  |  |
| **Change in LA area (cm2)** | **Baseline LA area** | -0.51 | 0.11 | -4.44 | <0.001 | -0.74 | -0.27 |  |  |
|  | **Vit D vs. Placebo** | -0.27 | 0.91 | -0.30 | 0.76 | -2.12 | 1.57 |  |  |
|  | **Constant** | 1.39 | 0.59 | 2.33 | 0.03 | 0.18 | 2.59 |  |  |

Legend: Multiple imputation analyses under MAR assumption.

Supplementary Figure S1: Contour enhanced funnel plots.

Legend: Contour enhanced funnel plots. A test for funnel plot asymmetry (sometimes referred to as a test for small study effects) examines whether the association between estimated intervention effects and a measure of study size is greater than might be expected to occur by chance. These tests typically have low power, so even when a test does not provide evidence of asymmetry, bias cannot be excluded. Here, there are no studies in the shaded areas of high significance hence the publication bias is unlikely to be the underlying cause of apparent asymmetry. (Sterne, J. et. al., Recommendations for examining and interpreting funnel plot asymmetry in meta-analyses of randomised controlled trials, BMJ 2011;342:d4002)

***Tables with raw data***

Table S5: Baseline raw data summary for clinical outcomes of interest

|  | **PLACEBO (N=23)** | | | **VITAMIN D (N=25)** | | |  |
| --- | --- | --- | --- | --- | --- | --- | --- |
|  | **MEAN (SD)** | **MEDIAN (Q1,Q3)** | **NO** | **MEAN ( SD)** | **MEDIAN (Q1,Q3)** | **NO** | **TEST** |
| **Baseline LV ED mass (g)** | 100.03(29.65) | 93.70(77.70,108.50) | 23 | 105.31(40.06) | 99.05(77.35,114.55) | 24 | 0.97 |
|  |  |  |  |  |  |  |  |
| **Baseline LV ESV (ml)** | 51.55(16.66) | 52.00(40.10,63.80) | 23 | 57.27(16.50) | 54.05(46.55,66.05) | 24 | 0.25 |
|  |  |  |  |  |  |  |  |
| **Baseline LV EDV (ml)** | 147.80(36.31) | 151.40(129.60,180.80) | 23 | 149.60(34.06) | 146.25(124.15,171.20) | 24 | 0.86 |
|  |  |  |  |  |  |  |  |
| **Baseline LV EF (%)** | 65.37(5.57) | 65.2(62.8,69.3) | 23 | 61.8(5.43) | 61.85(57.05,66.1) | 24 | 0.031 |
| **Baseline LA area (cm2)** | 21.11(3.86) | 20.40(19.10,23.80) | 23 | 22.10(4.74) | 22.50(18.30,25.35) | 24 | 0.67 |
|  |  |  |  |  |  |  |  |
| **Baseline RV EDV (ml)** | 156.65(42.48) | 148.70(124.60,194.50) | 23 | 158.25(49.51) | 149.05(123.50,176.05) | 24 | 0.85 |
|  |  |  |  |  |  |  |  |
| **Baseline RV ESV (ml)** | 67.33(26.49) | 63.40(47.00,78.50) | 23 | 67.05(35.73) | 56.75(50.55,68.05) | 24 | 0.55 |
|  |  |  |  |  |  |  |  |
| **Baseline RV SV (ml)** | 89.30(22.97) | 93.90(71.70,102.30) | 23 | 91.98(23.36) | 91.35(74.10,107.65) | 24 | 0.69 |
|  |  |  |  |  |  |  |  |
| **Baseline RV EF (%)** | 57.54(7.96) | 57.90(52.10,63.90) | 23 | 59.96(6.20) | 61.50(54.95,64.70) | 24 | 0.25 |
| **Baseline RA AREA (cm2)** | 19.87(5.04) | 20.70(16.90,23.20) | 23 | 19.96(3.24) | 19.80(17.50,22.50) | 24 | 0.94 |

Legend: The p-values correspond to appropriate two-independent sample tests (t-test or Kruskal Wallis after checking the normality assumption) which evaluated the generic null hypotheses of no difference between the samples’ distributions corresponding to the two treatment groups. LV ED=left ventricular end diastolic, ESV= end systolic volume, EF=Ejection fraction, LA=left atrial, RA=right atrial

Table S6: Follow up raw data summary for clinical outcomes of interest

|  | **PLACEBO (N=23)** | | | **VITAMIN D (N=25)** | | |  |
| --- | --- | --- | --- | --- | --- | --- | --- |
|  | **MEAN (SD)** | **MEDIAN (Q1,Q3)** | **NO** | **MEAN ( SD)** | **MEDIAN (Q1,Q3)** | **NO** | **TEST** |
| **Follow up LV ED mass (g)** | 95.99(26.84) | 93.65(84.1,105.3) | 22 | 108.54(38.98) | 101.4(76.6,128.4) | 18 | 0.28 |
|  |  |  |  |  |  |  |  |
| **Follow up LV ESV (ml)** | 52.59(17.84) | 50.95(41.30,60.50) | 22 | 58.81(15.59) | 56.90(50.10,71.10) | 18 | 0.25 |
|  |  |  |  |  |  |  |  |
| **Follow up LV EDV (ml)** | 148.92(35.96) | 151.35(124.30,175.00) | 22 | 153.75(35.57) | 157.55(129.20,176.10) | 18 | 0.67 |
|  |  |  |  |  |  |  |  |
| **Follow up LV EF (%)** | 65.22(5.62) | 64.55(62.2,71) | 22 | 61.79(4.71) | 60.75(58,65.3) | 18 | 0.043 |
|  |  |  |  |  |  |  |  |
| **Follow up LA area (cm^2^)** | 21.77(3.73) | 21.5(20.6,24.3) | 22 | 22.36(3.73) | 22.05(19.5,24.7) | 18 | 0.63 |
|  |  |  |  |  |  |  |  |
| **Follow up RV EDV (ml)** | 160.88(42.81) | 153.30(130.60,202.70) | 22 | 162.12(38.84) | 156.70(137.00,193.30) | 18 | 0.92 |
|  |  |  |  |  |  |  |  |
| **Follow up RV ESV (ml)** | 66.40(26.40) | 61.65(45.70,81.60) | 22 | 63.58(14.97) | 63.45(50.90,75.10) | 18 | 0.69 |
|  |  |  |  |  |  |  |  |
| **Follow up RV SV (ml)** | 95.64(24.56) | 95.85(72.80,111.30) | 22 | 98.51(33.02) | 90.85(72.40,130.20) | 18 | 0.75 |
|  |  |  |  |  |  |  |  |
| **Follow up RV EF (%)** | 59.94(7.02) | 60.45(55.00,64.50) | 22 | 59.85(8.66) | 62.30(53.70,66.90) | 18 | 0.97 |
| **Follow up RA AREA (cm^2^)** | 19.86(4.84) | 20.35(16.30,23.10) | 22 | 20.46(4.11) | 19.70(17.90,22.80) | 18 | 0.68 |

Legend: The p-values correspond to appropriate two-independent sample tests (t-test or Kruskal Wallis after checking the normality assumption) which evaluated the null hypotheses of no difference between the two treatment groups. . LV ED=left ventricular end diastolic, ESV= end systolic volume, EF=Ejection fraction, LA=left atrial, RA=right atrial
